# Major amputations in type 2 diabetes between 2001 and 2015 in Spain: regional differences

**DOI:** 10.1186/s12889-019-8137-7

**Published:** 2020-01-14

**Authors:** María del Cristo Rodríguez Pérez, Chiara Chines, Arturo J. Pedrero García, Djeniffer Sousa, Francisco J. Cuevas Fernández, Itahisa Marcelino-Rodríguez, Santiago Domínguez Coello, Antonio Cabrera de León

**Affiliations:** 1 Research Unit, Canarian Health Service, Ntra. Sra. de Candelaria University Hospital and Primary Care Authority, 38010 Santa Cruz de Tenerife, Spain; 2 Preventive Medicine and Public Health, Universidad de La Laguna, Santa Cruz de Tenerife, Spain; 3 Health Center of Primary Care Barranco Grande, Canary Health Service, Santa Cruz de Tenerife, Spain; 4 Health Center of Primary Care La Victoria, Canary Health Service, Santa Cruz de Tenerife, Spain

**Keywords:** Type 2 diabetes, Major amputation, Incidence, Mortality, Hospital stay

## Abstract

**Background:**

To analyze the trend of lower extremity major amputations (MA) among patients with type 2 diabetes mellitus (T2DM) in the Regions of Spain from year 2001 until 2015.

**Methods:**

Descriptive study of 40,392 MA. Data were obtained from the national hospital discharge database in patients with T2DM. The incidence rate was calculated in each Region, in addition to the incidence ratios (IR) between annual incidence and incidence of the year 2001. The length of hospital stay and mortality risks were analyzed using regression models adjusted for sex, age and smoking.

**Results:**

The major amputations incidence rate per 100,000 person-years was 0.48 in Spain; Canary Islands showed the highest incidence (0.81). The trend was a slight decrease or stability of the incidence in all Regions except in the Canary Islands (IR_2015_ = 2.0 [CI95% = 1.5, 2.6]) and in Madrid (IR_2015_ = 0.1 [CI95% = 0.1, 0.2]). Mortality after major amputations was 10% in Spain; Cantabria suffered the highest risk of death [1.7 (CI95% = 1.4; 2.1), *p* < 0.001] and La Rioja the lowest risk (0.5 [CI95% = 0.2; 0.9]; *p* = 0.026). The longest hospital stay was registered in the Canary Islands [(CI95% = 11.4;13.3], *p* < 0.001)], and the shortest in the Valencian Community [(CI95% = − 7.3; − 5.8), p < 0.001)].

**Conclusion:**

MA in T2DM followed a growing trend in the Canary Islands, which diverged from the downward trend in Spain. The variability of mortality and hospital stay, suggest to review the clinical management in some Regions. Sudden incidence decrease in Madrid suggests checking the record procedures of hospital discharges.

## Background

More than a half of the non-traumatic amputations of limbs occur in patients with type 2 diabetes (T2DM), although during the twenty-first century the incidence of these amputations has been decreasing in Europe, USA, and Asia [1–3]. Despite this decrease, major lower extremity amputations (MA) still remain a cause of high mortality and loss of quality of life with a significant increase in health costs associated to diabetes [2]. In recent decades there has been an improvement in the early detection and control of T2DM complications and, since the Saint Vincent Declaration in 1990, diabetic foot care has been intensified to reduce the incidence of major amputations. However, the increase in life expectancy of patients diagnosed with T2DM has also increased the associated morbidity [4]. Advanced age, African ethnicity and male sex are associated with an increased risk of amputations in patients with T2DM, while other factors such as female gender, kidney failure and congestive heart failure increase the risk of death during hospitalization [5]. In addition, undergoing a MA is a significant risk factor for long term mortality among T2DM patients [6].

The overall incidence of amputations due to diabetes increased by 4.29% in Spain between 2001 and 2004, followed by a downward trend until 2012, around 2% [7]. In-hospital mortality due to amputations remained stable between 2001 and 2012, around 10%, resulting in an economic cost of 33% of the total cost spent in hospital treatment of T2DM complications [8]. Although there are several published works in relation to non-traumatic amputations in Spain [5–8], they account for the major and minor procedures globally in the analysis, or do not differentiate the type of diabetes. To our knowledge, there are no publications that have compared MA incidence in patients with T2DM in the seventeen Autonomous Communities (AACC) or Regions of Spain, nor analyzed possible regional heterogeneity in MA mortality or days of hospital stay.

We conducted the present study in order to analyze the trend of major amputations incidence in patients with T2DM during the period 2001–2015 in all the AACC of Spain, as well as mortality and hospital stay in each of them.

## Methods

### Design and subjects

This is a retrospective observational study of the Spanish National Hospital Discharges of 40,392 hospital admissions for major amputations (defined as through, or proximal to, the tarsometatarsal joint) in patients with T2DM in the seventeen AACC of Spain, during the period 2001–2015. Data were collected from the Minimum Basic Data Set (CMBD, in Spanish) obtained from public and private hospitals. CMBD is a record of clinical-administrative data integrated into the health information system of the national health system, and contains the information at discharge of all episodes of hospitalization in the country. The database for this study was produced by the Ministry of Health with aggregated fully anonymized data, so neither individual written consent by patients nor ethical approval was required because in Spain the use of the CMBD does not require prior approval of an ethics committee (under the Spanish Organic Law 3/2018 on the protection of personal data and digital rights, which transcribe the European Union General Data Protection Regulation 2016/679).

The information was obtained from the principal and secondary diagnoses for each episode of admission in addition to age, sex and hospital code, current consumption of tobacco, admission date and causes for hospital discharge (including death). The database did not include other socio-economic or lifestyle data. The length in days of hospital stay in each AACC was also analyzed, and its difference was calculated with respect to the average stay in Spain.

#### Inclusion and exclusion criteria

Only cases of MA in patients with T2DM were selected (ICD9-CM codes ranging from 250.00 to 250.92, except codes for type 1 diabetes). The records of patients under 15 years of age were excluded because there were not MA patients with T2DM under this age. We also excluded those cases in which sex or geographical area had not been recorded, as well as episodes recorded in the North-African cities of Ceuta and Melilla.

### Statistical analysis

The scale variables were summarized according to their mean and standard deviation, or by percentiles. The categorical variables were presented by the observed frequency (%) and the 95% Confidence Interval (CI95%).

The MA incidence rates during the period 2001–2015 were computed for the population in each Region and in the whole of Spain; for each geographical area the numerator was the sum of cases in this period in patients of 15 or more years of age, and the denominator was the sum of all the resident annual populations with that same age, obtained from the National Institute of Statistics (Spain). These regional MA rates were age-standardized by the direct method, taking as a reference the population of Spain.

In addition, in each Region the ratio between the annual incidence of MA respect to the incidence of 2001 was calculated, and the Open-Epi package was used to obtain the CI95% of these incidence ratios (IR). The trend of the IR was analyzed with a sequence chart to plot time series.

Linear regression models were used to adjust for age, sex and smoking the differences in the length of hospital stay. Regression coefficients and their confidence intervals are offered with a CI95%. To estimate the risk of mortality after MA, logistic regression models were adjusted for sex, age and active smoking; Odds ratios (OR) and their CI95% were obtained in this way.

Statistical analyses were performed using the statistical package SPSS 21.0, in Spanish.

## Results

**Fig. 1:**
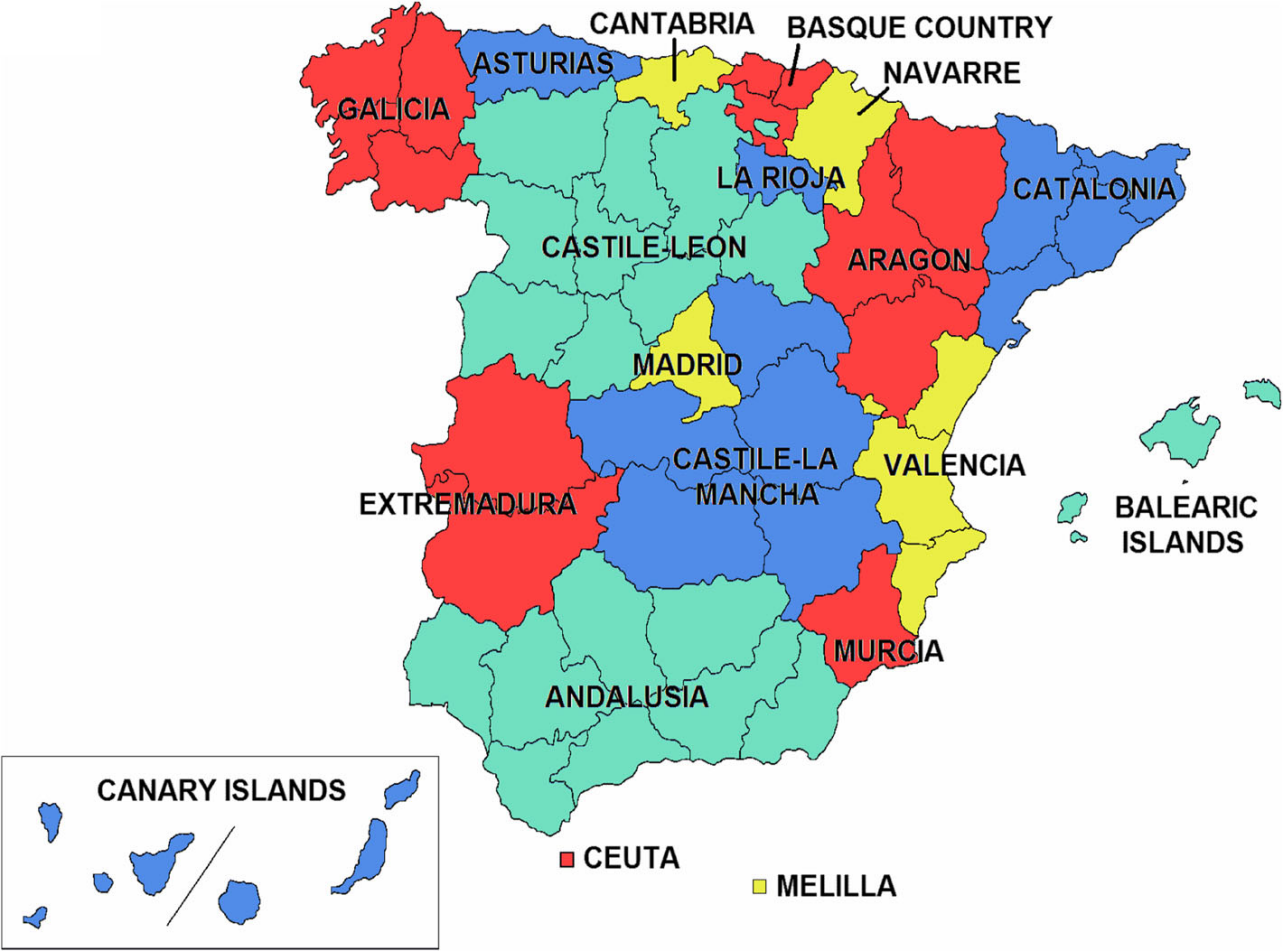
The seventeen Autonomous Communities or Regions of Spain (free distribution under Creative Commons license. https://commons.wikimedia.org/wiki/File:Ccaa-spain.png)

The study included 40,392 non-traumatic MA in patients with T2DM in the seventeen AACC of Spain (Fig. 1) during the period 2001–2015.

Table 1 shows the number of cases in each Region, as well as their age, proportion of women, active smokers, days of hospital stay and mortality derived from MA procedures. The average age of patients who underwent MA was above 72.5 years in all the AACC, except in the Balearic and Canary Islands. Among them, the prevalence of active smokers varied between 12% in Navarra and 5% in the Basque Country or La Rioja. The Regions with the longest hospital stay for MA were Aragon, Asturias and The Canaries; by contrast, the Balearic Islands, the Valencian Community and Murcia were the Regions with the shortest stay. Regarding mortality after MA, Cantabria showed the highest rate (17%) and La Rioja the lowest one (5%).

**Table 1:** Frequency of major amputations, average age, proportion of women, prevalence of active smoking, average length of hospital stay, and mortality rates in patients with T2DM in each Region, during the period 2001–2015

	Cases n (%)	Age X ± DE	Women %	Smoking %	Days of Stay P50 (P25; P75)	Mortality %
ANDALUSIA	9417 (23.3)	73.1 ± 10.2	36.6	9.1	14 (8;24)	10.1
ARAGON	996 (2.5)	75.1 ± 10.4	34.8	10.0	26 (17;44)	14.0
ASTURIAS	1280 (3.2)	74.6 ± 10.6	38.0	7 .0	24 (15;39)	8.1
BALEARICS	808 (2)	72.1 ± 10.8	34.0	9.8	13 (8;25)	9.0
CANARY ISLANDS	2328 (5.8)	72.1 ± 11.1	35.2	10.0	23 (13;41)	8.1
CANTABRIA	729 (1.8)	76.1 ± 10.4	40.9	9.2	22 (13;37)	16.6
CASTILLA AND LEON	1886 (4.7)	76.8 ± 9.8	34.9	5.7	18 (11;29)	11.4
CASTILLA LA MANCHA	1611 (4)	74.4 ± 10.4	36.4	10.4	16 (9;27)	9.5
CATALONIA	6352 (15.7)	73.3 ± 10.6	35.5	6.4	18 (10;30)	9.1
VALENCIAN COMMUNITY	4446 (11)	72.6 ± 10.5	35.1	10.0	14 (8;22)	8.4
ESTREMADURA	1198 (3)	74.5 ± 10.3	37.1	5.4	16 (10;27)	10.1
GALICIA	2942 (7.3)	74.5 ± 10.4	38.3	6.9	19 (11;34)	11.7
MADRID	2520 (6.2)	73.7 ± 11.1	38.3	9.0	20 (12;34)	11.9
MURCIA	1576 (3.9)	73.2 ± 10.2	37.2	7.7	14 (9;23)	11.2
NAVARRE	397 (1)	76.0 ± 9.6	29.5	11.6	18 (11;31)	14.1
BASQUE COUNTRY	1733 (4.3)	73.7 ± 10.3	30.7	4.5	17 (11;28)	6.7
RIOJA	173 (0.4)	75.9 ± 9.2	34.7	5.2	15 (10;24)	5.2
SPAIN	40,392 (100.0)	73.6 ± 10.5	36.1	8.2	17 (10;29)	10.0

Table 2 presents the ratios between the annual incidence of MA and the incidence for the initial year (2001), showing a strong increase in the Canary Islands ratio (IR = 2.0 at the end of the 15 years period), compared to the moderate decrease in the whole country (IR = 0.8). The greatest decrease occurred in the Region of Madrid (IR = 0.1 at the end of the period [CI95% = 0.08; 0.17]), but it should be noted that the incidence of this Community experienced a very sharp fall (greater than 80%) between 2009 and 2010. This table also shows the MA incidence rates in the AACC for the entire period; the Canarian rate (0.81 / 100,000 people-year) was almost double that of Spain incidence rate (0.48 / 100,000 people- year) and triple that of Madrid (0.24 / 100,000 people-year).

**Table 2:** Ratios between the annual incidence of major amputation and the initial year (2001) incidence of the studied period; CI95% is offered for ratios in the final year of the period (2015). Major amputation incidence rates for the period 2001 to 2015 per 100,000inhabitants * year-1

	2001	2002	2003	2004	2005	2006	2007	2008	2009	2010	2011	2012	2013	2014	2015 (CI95%)	Period Incidence
ANDALUCIA	1.00	0.93	1.02	1.09	1.08	1.09	1.05	0.97	0.98	0.84	0.98	0.98	0.94	0.90	0.94 (0.84;1.06)	0.71
ARAGON	1.00	1.02	1.01	1.03	1.41	1.29	1.00	1.13	1.46	1.31	1.01	1.21	1.28	1.16	1.03 (0.71;1.49)	0.34
ASTURIAS	1.00	0.97	0.80	0.96	0.61	0.93	1.05	1.10	0.89	0.72	0.51	0.43	0.39	0.57	0.59 (0.43;0.80)	0.48
BALEARICS	1.00	1.04	1.42	1.15	1.27	1.16	1.11	0.98	1.06	1.06	0.96	0.70	0.69	0.63	0.52 (033;0.82)	0.49
CANARY ISLANDS	1.00	1.25	1.37	1.63	1.68	1.69	1.74	1.76	1.89	1.72	1.84	1.97	1.57	2.05	2.00 (1.54;2.59)	0.81
CANTABRIA	1.00	0.95	1.30	1.47	1.52	1.13	1.16	1.41	1.07	0.81	0.89	0.47	0.83	0.58	0.36 (0.21;0.62)	0.59
CASTILLA AND LEON	1.00	1.02	0.85	0.90	1.07	1.02	1.15	1.05	1.10	0.91	1.05	0.93	0.95	0.88	0.84 (0.65;1.09)	0.29
CASTILLA LA MANCHA	1.00	1.04	1.17	0.99	0.88	1.27	0.95	1.16	1.09	0.79	0.86	0.85	0.81	0.94	0.84 (0.64;1.12)	0.40
CATALONIA	1.00	0.99	1.43	1.35	1.30	1.26	1.15	1.26	1.18	0.88	0.90	0.95	0.88	0.91	1.09 (0.94;1.26)	0.47
VALENCIAN COMMUNITY	1.00	1.08	1.05	1.23	1.04	1.17	1.26	1.17	1.17	1.00	1.04	0.92	0.88	0.80	0.80 (0.67;0.96)	0.50
ESTREMADURA	1.00	1.16	0.96	1.05	1.17	1.10	1.39	1.10	1.12	0.87	0.72	0.50	0.83	0.84	0.75 (0.54;1.05)	0.53
GALICIA	1.00	1.16	1.09	1.12	1.06	1.19	1.18	1.14	1.16	0.88	0.72	0.77	0.75	0.67	0.78 (0.63;0.97)	0.44
MADRID	1.00	1.23	1.09	1.06	1.12	1.14	1.18	1.07	1.07	0.19	0.18	0.25	0.21	0.18	0.11 (0.08;0.17)	0.24
MURCIA	1.00	1.09	1.07	1.21	1.16	1.18	1.28	1.20	1.21	0.96	1.15	0.89	1.17	0.99	0.80 (0.59;1.08)	0.72
NAVARRE	1.00	0.90	1.04	1.24	1.48	0.75	1.13	1.21	1.05	0.84	0.73	0.30	0.76	0.67	0.87 (0.50;1.50)	0.32
BASQUE COUNTRY	1.00	1.03	0.78	0.88	0.87	0.93	0.86	0.91	0.85	0.76	0.82	0.69	0.63	0.59	0.44 (0.32;0.59)	0.38
RIOJA	1.00	0.55	0.87	0.66	0.64	0.95	0.69	0.24	1.14	0.90	0.54	0.48	0.36	0.67	1.22 (0.61;2.41)	0.26
SPAIN	1.00	1.04	1.09	1.13	1.11	1.15	1.13	1.11	1.10	0.85	0.87	0.84	0.82	0.81	0.80 (0.75;0.84)	0.48

Table 3 shows the multivariate adjustment of the length of hospital stay between each community and the total of Spain, as well as the risk of death in each Region compared to the overall mortality in Spain. Both models were adjusted for sex, age and smoking; they corroborated that the longest hospital stay by MA occurred in the Canary Islands, being 12 days longer than the average of the country and 19 days longer than the Region with the shortest stay (Valencian Community). However, the risk of in-hospital death after MA was higher in Cantabria, which exceeded the overall risk in Spain by 70%, while in La Rioja there was a 50% reduction in the risk of death with respect to the country.

**Table 3:** Difference (CI95%) between days of hospital stay for major amputation respect to the global of Spain in patients with type 2 Diabetes. Risk (CI95%) of death in-hospital following major amputation compared to Spain in patients with type 2 Diabetes. Results adjusted for age, sex and active smoking in each Region

	Difference of Stay	Risk of Death
ANDALUCIA	−5.4 (− 5.9; − 4.8); *p* < 0.001	1 (0.9; 1.1); *p* > 0.05
ARAGON	11.9 (10.5; 13.5); *p* < 0.001	1.4 (1.2; 1.7); *p* < 0.001
ASTURIAS	8.2 (6.9; 9.6); p < 0.001	0.8 (0.6; 0.9); *p* = 0.010
BALEARICS	−2.9 (−4.6; −1.3); p < 0.001	0.9 (0.7; 1.2); p > 0.05
CANARY ISLANDS	12.4 (11.4; 13.3); p < 0.001	0.8 (0.7; 0.9); *p* = 0.014
CANTABRIA	7.7 (5.9; 9.4); p < 0.001	1.7 (1.4; 2.1); p < 0.001
CASTILLA AND LEON	1.3 (0.2; 2.4); *p* = 0.021	1.1 (0.9; 1.2); p > 0.05
CASTILLA LA MANCHA	−1.8 (−2.9; −0.6); *p* = 0.004	0.9 (0.8; 1.1); *p* > 0.05
CATALONIA	0.1 (−0.5; 0.8); p > 0.05	0.9 (0.8; 0.9); *p* = 0.017
VALENCIAN COMMUNITY	−6.6 (−7.3; −5.8); p < 0.001	0.8 (0.7; 0.9); p = 0.003
ESTREMADURA	−2 (−3.4; −0.7); *p* = 0.003	0.9 (0.8; 1.2); p > 0.05
GALICIA	5.6 (4.8; 6.5); p < 0.001	1.2 (1.1; 1.3); *p* = 0.006
MADRID	4.3 (3.3; 5.2); p < 0.001	1.2 (1.1; 1.4); *p* = 0.001
MURCIA	−5.3 (−6.5; −4.1); p < 0.001	1.2 (0.9; 1.4); p > 0.05
NAVARRE	2.8 (0.5; 5.2); *p* = 0.019	1.4 (1.1; 1.9); p = 0.014
BASQUE COUNTRY	−1.6 (−2.8; −0.5); p = 0.006	0.6 (0.5; 0.8); p < 0.001
RIOJA	−4.4 (−7.9; −0.8); *p* = 0.016	0.5 (0.2; 0.9); p = 0.026

**Fig. 2:**
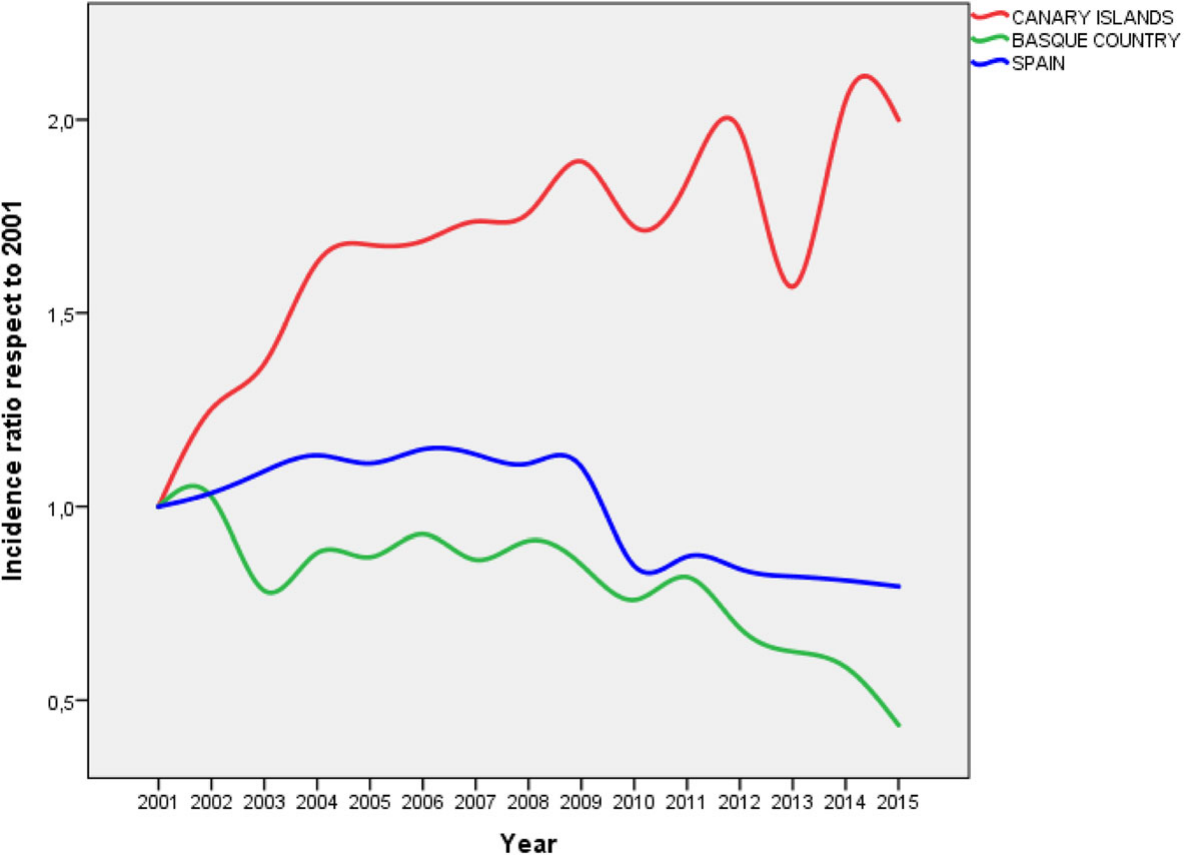
Trend of the incidence ratio of major amputation in patients with T2DM in Spain, Canary Islands and the Basque Country for each year of the period in relation to 2001

Figure 2 represents the trend of the annual incidence ratios compared to 2001 for two AACC of similar population size (The Canary Islands and the Basque Country) and for the global of Spain.

## Discussion

The study of 40,392 hospital admissions for nontraumatic major amputations occurred in patients with T2DM in Spain during the period 2001–2015 showed a high variability among AACC, both in incidence and length of hospital stay and mortality. The trend of the incidence has been moderately decreasing in the whole of the country, but more accentuated in the Community of Madrid; however, the exception was the Canarian Community, that has worsened until doubling its incidence in respect to the initial years of the data reviewing period.

Although T2DM mortality has declined in these islands significantly in the last five years, [9] the severity of this disease in the Canary Islands is known; its current population suffers the highest incidence and prevalence [10, 11] in Spain, as well as high mortality rates due to this disease [12]. It is also known the high incidence of MA in Canarian population [13] but the trends with respect to the other Regions had not been studied. We have corroborated that, far from approaching the improvement experienced in the whole of the country, the trend of the MA in the Canary Islands diverged from the other AACC during this century. At the opposite extreme is the Community of Madrid, which is known to have the lowest prevalence and mortality by T2DM in Spain [10] and for which we have now found the best trend in the country for MA incidence; nevertheless, we want to draw attention to the sudden decline of MA in this Region as of 2009, which could be attributed to an error in the record procedures of hospital discharges.

Looking at the population size, the most similar Region to the Canaries is the Basque Country, whose MA prevalence in T2DM had been previously studied [14]. In both AACC the population aged over 15 years (1,804,000 inhabitants) represented 4.7% of the Spanish population in 2015 [15], but for the studied period their cases of MA were 5.8% in the Canary Islands (that is, 23% more than expected) and 4.3% in the Basque Country (that is, 9% less than expected); so, the divergent trend produced a ratio of 4.5 between their incidence rates for 2015/2001. In addition, according to previous publications [2, 4, 7] the general profile of MA patients, in order to age and gender, corresponded to a 74-year-old male; but in the Canary Islands the average age of the patients had underwent a MA was 1.5 years younger than in Spain. This increases the burden of T2DM in Canarian population in terms of lost years of potential life free of disability.

In European and North-American countries there has been a decrease in the incidence of nontraumatic amputations in the last decades, [16] especially at the expense of MA in patients with T2DM [17] and particularly more evident for major amputations above the knee [18]; this has been attributed to the healthcare offered to the patient with T2DM, specially the diabetic foot care programmes [4]. This decreasing trend was also reported in Spain [7, 19]. However, most studies do not analyze regional differences, as our study does. Our analysis questions the homogeneity of the Spanish health system and suggests the need for further research on the causes of the detected variability. Some aspects like the identification of access barriers, and the provision of early revascularization services must be taken into account to improve the diabetic foot care programmes [20].

The great variability that our study detected in the length of hospital stay due to MA reveals an important heterogeneity of clinical management within the National Spanish Health System. The Canarian and Valencian Communities presented the extreme data points, with 19 days of difference between them for an average hospital stay. We have not used in this article the costs provided by the CMBD because they are estimates for the entire health system and its disaggregation by AACC would not represent the real costs. Undoubtedly, further duration of the hospital stay increases the costs of the procedures and, in addition, negatively affects the beds availability and increases the risk of nosocomial infections too. According to this, we believe that length of hospital stay management should be reviewed, especially in the Regions with the longest hospital stay, pointing out after Canary Islands the cases of Aragon and, at some distance, Cantabria.

Looking at mortality during hospitalization for MA, we have found another important difference among Regions, where the highest mortality risk (Cantabria) tripled the lowest risk (La Rioja and Basque Country). These results were not modified when the Community of residence was used instead of the Community of hospitalization, nor when they were adjusted for length of stay, age, sex or smoking. It could be speculated that MA criteria in T2DM could be more conservative in some AACC than in others; in fact, the seven AACC with the highest risk of death had a lower incidence of MA than the average of the country. Once again, we believe that Regions with the highest mortality risks should review the clinical management of MA.

The prevalence of smoking in the studied population exceeded 10% in some AACC. Certainly, smoking was higher in the general adult population of Spain, [21] but it should be noted that this 10% refers to active smoking in patients with T2DM admitted for severe macrovascular complications. We cannot demonstrate that these patients had been informed that tobacco increases the risk of amputations and, in addition, suffering an amputation does not implies smoking cessation [22]. New approaches are needed to ensure the effectiveness of tobacco control policies.

The main limitation of our study is that data came from clinical-administrative hospital records and there is a possibility of under-registration of T2DM as well as an underestimation of incidence of non-traumatic amputations in those cases. In addition, it remains difficult to establish a causal relationship that explains the differences detected regarding mortality in the Regions due to the lack of relevant information such as the number of years having T2DM, concomitant treatments or disease control status. Systematic reviews of studies on the incidence of amputations indicate that the variability detected could be explained by aspects related to the design, statistical methods used or the heterogeneity in the definition of the variables [23]. Despite this, the CMBD has shown a confident quality of data and a valid exploitation for health research [24]. In contrast, the main strength of our study is the large sample size, which includes patients admitted to hospitals for MA in whole of Spain.

## Conclusions

We conclude that the trend of MA incidence was moderately favorable for the global of Spain during the first fifteen years of this century with the only exception of the Canary Islands, which increased its incidence. The great variability in mortality and length of hospital stay due to MA questions the homogeneity of the healthcare provided by the National Spanish Health System. The Regions with worse outcomes should make efforts to identify the reasons for the observed differences and improve the healthcare quality. The abrupt decline of MA in the Community of Madrid after 2009, advises to revise the record procedures of its hospital discharges. More national and regional studies monitoring nontraumatic amputations in patients with T2DM are needed.

## Data Availability

All raw data on which our study is based can be accessed from the Ministry of Health (icmbd@msssi.es).

## Abbreviations

AACC: Autonomous Communities
IR: Incidence ratio
MA: Lower extremity major amputations
T2DM: Type 2 diabetes mellitus
